# Issues and Challenges Associated with Data-Sharing in LMICs: Perspectives of Researchers in Thailand

**DOI:** 10.4269/ajtmh.19-0651

**Published:** 2020-05-11

**Authors:** Jaranit Kaewkungwal, Pornpimon Adams, Jetsumon Sattabongkot, Reidar K. Lie, David Wendler

**Affiliations:** 1 Department of Tropical Hygiene, Faculty of Tropical Medicine, Mahidol University, Bangkok, Thailand;; 2 Office of Research Services, Faculty of Tropical Medicine, Mahidol University, Bangkok, Thailand;; 3 Mahidol Vivax Research Unit, Faculty of Tropical Medicine, Mahidol University, Bangkok, Thailand;; 4 Department of Philosophy, University of Bergen, Bergen, Norway;; 5 Department of Bioethics, National Institutes of Health Clinical Center, National Institutes of Health, Bethesda, Maryland

## Abstract

Data-sharing helps advance scientific research and assures the benefits of research data are maximized. Previous work has highlighted ethical challenges, especially in low- and middle-income countrie (LMIC) countries. This study examined the views of researchers in a middle-income country, Thailand, regarding the most important data-sharing challenges. The target researchers worked in biomedical and related research. The survey was distributed to 38 academic and health-science institutes, 18 university hospitals, 84 nonuniversity hospitals, and 22 research institutes across Thailand; 229 researchers in clinical/basic and social/behavioral sciences, and pubxxlic health/policy participated. Thai researchers were less concerned with informed consent and the feasibility of conducting research and sharing data, focusing on the importance of safeguards when handling data, including transfer to others, and possible lack of control over subsequent data use. The respondents felt that researchers should decide what types of project data are shareable and which data are likely useful to the scientific community. They were more concerned with appropriate acknowledgment and protecting the legal rights of the primary data collectors and providers. Although they had concerns about data access conditions, they rated sharing sufficient data and metadata to reproduce the analysis of the primary outcomes as highly important. These results are important for future efforts of the LMIC countries to develop efficient data-sharing frameworks and establish institutional data access committees. They highlight the importance, for the sustainability and fairness of these efforts, to ensure that parties in LMIC countries receive appropriate credit and are involved in determining where/when/how their data may be used.

## INTRODUCTION

Sharing data from clinical and public health research has the potential to generate more and better science, and to enable more efficient use of research resources.^1–3^ For these reasons, sharing data has become a core requirement for biomedical research.^1,4^ The Council for International Organizations of Medical Sciences (CIOMS) states that “researchers, sponsors, and research ethics committees (RECs) must share data for further research where possible.”^5^ Sharing research is also regarded as best practice by the WHO and other professional organizations.^6^

Although data-sharing is valuable, it raises important cultural, ethical, financial, and technical challenges.^2,7,8^ These include finding the right balance between making data accessible and safeguarding privacy, ensuring access, determining authorship, and protecting intellectual property.^2,9^ A study on global data-sharing expressed concern that these challenges may be greater in low- and middle-income countries (LMICs) because of perceived disparities in decision-making between primary data producers and secondary data users.^2^ Another study by a research unit in Thailand that hosts a research data repository noted that very few researchers in LMICs have requested data for secondary analyses and that most applications for secondary data use came from high-income countries.^10^ If challenges related to informed consent, data management, data dissemination, and validation of research contributions are more difficult in LMICs, it would raise concerns over the appropriateness of encouraging and requiring data-sharing in these settings.^2^

As a background to the practical and ethical frameworks for data-sharing in Thailand, the regulations and guidelines regarding sharing health-related data in Thailand comply with international standards. The Medical Profession Act BE 2525 (AD 1982), the Medical Council Regulations on Medical Ethics Preservation BE 2549 (AD 2006) I/IV, and the National Health Act BE 2550 (AD2007) II/II provide the definitions of medical practice and research; there were a few statements about data ownership and the use of data for treatment, care, and research purposes.^11–13^ The National Health Act stated that health data, being personal and confidential information, cannot be released at the risk of causing damage in the absence of the person’s consent, and violation could lead to 6-month imprisonment and/or a 10,000 baht (approximately US$300) fine; the offense, however, can be compoundable. When they review proposals, RECs across Thailand use several other national guidelines, which generally contain few statements on data- and specimen-sharing, including Ethical Guidelines for Research on Human Subjects in Thailand (2007), National Policy and Guidelines for Human Research (2015), and the Guidance in Clinical Trial Safety Information among Stakeholders (2011).^14^ Although several revisions of the first Human Research Act have been an ongoing process since 1985, a new “Personal Data Protection Act BE 2562” was proclaimed in 2019. This act contains intensive details of the roles and responsibilities of data producers, data holders, and data users, like the European General Data Protection Regulation (GDPR).^15,16^ This regulation changed certain national ethical practices; for example, informed consent now could be provided electronically if written consent were not practicable.

In Thailand, the Ministry of Public Health launched the eHealth strategy in 2017, covering policy and guidelines related to the use of health-related data at the national level.^17^ Currently, the National Research Council of Thailand and Thailand Research Organizations Network are attempting to establish the Thai National Research Repository (TNRR),^18^ but the process is lengthy. The data-sharing of basic demographic and health-related data has been actively practiced by a few governmental organizations, for example, the Bureau of Epidemiology, National Statistical Office, and National Health Security Office.^19–21^ With their national roles and responsibilities in data consolidation and as data providers, the two offices hold a very large repository of health-related data for the country. The offices have their own policies and operating procedures for sharing the data in their databases; however, no formal data access committee (DAC) was established. A few consortia, networks, and research groups in Thailand have attempted to collect data for sharing. The Thai Medical Research Foundation has developed a website called the “Data Archival for Maximum Utilization System,” to collect databases/registries.^22^ Another group, the Ganesh SAP Research Unit, developed a database to collect health technology assessment information, including published studies, reports, theses, and proceedings conducted in Thailand.^23,24^ To the best of our knowledge, no formal data access committees (DACs) have been established and operationalized by institutional-level Thai research groups. The Mahidol-Oxford Tropical Medicine Research Unit, as part of a Thailand-based collaborative research group, established its own DAC to manage their research data and oversee data-sharing of their research studies, along with a data-sharing policy and platform in their research settings within Thailand and elsewhere.^10^

A study of the healthcare data situation in six selected economies in the Asia–Pacific region (Thailand, PDR of China, South Korea, Taiwan, Japan, and Malaysia) reported that the six countries are quite similar regarding health-related data issues, even though their economic structures and population sizes differ.^25^ The primary objective of collecting healthcare data in these countries is to aid policymakers and researchers in policy decision-making. Regarding data-sharing in Thailand, like other countries in the region, the sharing of healthcare databases remains limited because of the fragmented nature of financing and healthcare service provision. In all six countries, data are accessible mostly in aggregated format, usually published on the website and in printed reports, making it difficult and time consuming for researchers to analyze; although researchers can request individual-level data, it is not always permitted.^25^ Another study reviewed healthcare databases in Thailand and Japan; based on 20 databases from Thailand, all were national governmental databases including surveillance/registries of population-based and individual-based information on population health status.^26^ The study also posed unresolved issues about database accessibility, and data-sharing and usability.^26^ In addition to issues surrounding the accessibility and usability of national databases, researchers may also have to deal with ethical issues and challenges in sharing data collected from their research studies. There are few empirical data available to assess these concerns. The present study thus aimed to evaluate the views of researchers about data-sharing in a middle-income country, Thailand. In particular, we assessed researchers’ views of the most important challenges related to data-sharing in Thailand.

## MATERIALS AND METHODS

### Questionnaire development.

A previous systematic literature review identified six categories of potential barriers to data-sharing: technical, motivational, economic, political, legal, and ethical.^9^ This review also identified specific concerns within each category. Based on this work, we developed our survey. The first section of the survey assessed the following 12 domains (see Supplemental Appendix for verbatim questions per domain): data covered, restriction on use, broad consent, modes of data-sharing, data documentation, data discoverability, data access conditions, data availability, timeliness of data-sharing, ethical issues, cost, and acknowledgment.

The second section of the survey asked respondents to rate the perceived difficulty or burden associated with sharing their research data: necessary resources (time and money), technical issues (data-sharing platforms, data management, and interoperable systems), issues related to proprietary data, issues related to ethical and legal compliance in sharing individual data, organizational/institutional policies for data-sharing, organizational/institutional services or supports to perform data-sharing, quality and integrity of shareable data (e.g., complete and homogeneous), control of the use of “sensitive” or “restricted” data by other researchers, citation of the dataset (original work), and acknowledgment of the data repository. Respondents were asked to rate the level of importance or difficulty of each issue on a Likert scale from 1 to 5, with 1 indicating least important/least problematic and 5 indicating highly important/most problematic.

The final version of the questionnaire provided definitions of “research data” and “data-sharing.” The questionnaire was developed in both English and Thai, as the respondents included Thai and non-Thai researchers in different organizations (see Supplemental Questionnaire File in attachment). The dual-language questionnaire was cross-validated by a native English speaker.

### Data collection.

The target respondents were researchers who had been working in biomedical and health-related research at universities, nonuniversity hospitals, and research institutes. Paper-based and online versions of the survey were developed. The online survey was distributed via e-mails that contained a link to the questionnaire. The paper-based and online versions of the survey were distributed to 218 participants from 38 academic and health-science institutes across Thailand, who were participating in a 2018 workshop on human research studies organized by the Office of Research Services, Faculty of Tropical Medicine (FTM), Mahidol University, Thailand. The online version was subsequently sent to the heads of the research offices at 18 university hospitals, 84 nonuniversity hospitals, and 22 research institutes, as well as alumni and researchers who had previously submitted proposals to and/or participated in workshops conducted by FTM. In total, 2,656 e-mails were sent from FTM. In addition, recipients of the online survey were asked to forward it to colleagues in their field.

Recipients were informed that completing the survey was voluntary. The survey was anonymous and not linked to the submitting source. Completed surveys were uploaded automatically to a database.

### Data analysis.

Descriptive statistics were presented as frequency and percentage by respondent’s field of work: clinical, basic science, social/behavioral, and public health/policy. Rating-level comparisons were evaluated using chi-square tests, with a *P*-value of < 0.05 considered statistically significant.

### Ethics clearance.

The Ethics Committee of the FTM, Mahidol University, Thailand, approved this study. Respondents were informed about the study purpose and told that participation was voluntary. They answered the questionnaire anonymously and were free to skip items they did not wish to answer.

## RESULTS

### Characteristics of the survey respondents.

A total of 229 respondents completed the survey. Based on the 2,656 surveys known to have been distributed, this suggests an overall response rate of 8.6% (229/2,656). As shown in Table 1, the respondents’ primary fields of research comprised the following: 123 (53.7%%) clinical study, 62 (27.1%) basic science/laboratory study, 15 (6.5%) social/behavioral science study, and 29 (12.7%) public health and policy study. About two-thirds were female. Most of the respondents worked for more than 15 years, particularly those in social and behavioral science and public-health areas.

**Table 1:** Characteristics of the survey respondents (*N* = 229)

Characteristic	Total, *n* (%)	Clinical, *n* (%)	Basic science, *n* (%)	Social/behavioral, *n* (%)	Public health/policy, *n* (%)
Gender	(*N* = 229)	(*N* = 123)	(*N* = 62)	(*N* = 15)	(*N* = 29)
Male	74 (32.3)	35 (28.5)	22 (35.5)	2 (13.3)	15 (51.7)
Female	155 (67.7)	88 (71.5)	40 (64.5)	13 (86.7)	14 (48.3)
Years of working	(*N* = 223)	(*N* = 119)	(*N* = 61)	(*N* = 14)	(*N* = 29)
1–3	41 (18.3)	25 (21.0)	12 (19.7)	1 (7.1)	3 (10.3)
4–6	35 (15.7)	17 (14.3)	12 (19.7)	1 (7.1)	5 (17.3)
7–10	34 (15.3)	20 (16.8)	7 (11.5)	5 (35.7)	2 (6.9)
10–15	34 (15.3)	25 (21.0)	4 (6.5)	0 (0.0)	5 (17.2)
> 15	79 (35.4)	32 (26.9)	26 (42.6)	7 (50.0)	14 (48.3)

### Perceived importance of issues in data-sharing.

As expected, the responses regarding the importance of the listed items skewed toward the upper range of the scale; that is, most of the items were rated as very important (4) or important (3). As shown in Table 2, more than 70% of all respondents rated as very important issues of ethics and acknowledgment, whereas less than half rated as very important issues related to data discoverability, timeliness of data-sharing, and cost.

**Table 2:** Perceived importance of issues related to data-sharing, by research field (*N* = 229)

Issues in data-sharing	Total (*N* = 229)			Clinical (*N* = 123)			Basic science (*N* = 62)			Social/behavioral (*N* = 15)			Public health/Policy (*N* = 29)			*P*-value
	Most important, *n* (%)	Important, *n* (%)	Less important, *n* (%)	Most important, *n* (%)	Important, *n* (%)	Less important, *n* (%)	Most important, *n* (%)	Important, *n* (%	Less important, *n* (%)	Most important, *n* (%)	Important, *n* (%)	Less important, *n* (%)	Most important, *n* (%)	Important, *n* (%)	Less important, *n* (%)	
Ethical issues	177 (77.3)	48 (21.0)	4 (1.7)	91 (74.0)	29 (23.6)	3 (2.4)	47 (75.8)	14 (22.6)	1 (1.6)	13 (86.7)	2 (13.3)	0 (0.0)	26 (89.7)	3 (10.3)	0 (0.0)	0.61
Acknowledgment	170 (74.3)	47 (20.5)	12 (5.2)	87 (70.7)	31 (25.2)	5 (4.1)	45 (72.6)	13 (21.0)	4 (6.4)	13 (86.6)	1 (6.7)	1 (6.7)	25 (86.2)	2 (6.9)	2 (6.9)	0.31
Data documentation	151 (65.9)	71 (31.0)	7 (3.1)	83 (67.5)	38 (30.9)	1 (1.6)	37 (59.7)	22 (35.5)	3 (4.8)	11 (73.3)	4 (26.7)	0 (0.0)	20 (69.0)	7 (24.1)	2 (6.9)	0.57
Restriction of use	146 (63.8)	76 (33.2)	7 (3.0)	77 (62.6)	41 (33.3)	5 (4.1)	44 (71.0)	18 (29.0)	0 (0.0)	10 (66.6)	4 (26.7)	1 (6.7)	15 (51.7)	13 (44.8)	1 (3.5)	0.44
Data access conditions	145 (63.3)	73 (31.9)	11 (4.8)	76 (61.8)	42 (34.1)	5 (4.1)	44 (71.0)	16 (25.8)	2 (3.2)	9 (60.0)	5 (33.3)	1 (6.7)	16 (55.2)	10 (34.5)	3 (10.3)	0.63
Data covered	138 (60.2)	73 (31.9)	18 (7.9)	68 (55.3)	42 (34.1)	13 (10.6)	42 (67.7)	19 (30.7)	1 (1.6)	11 (73.3)	3 (20.0)	1 (6.7)	17 (58.6)	9 (31.0)	3 (10.3)	0.33
Data availability	125 (54.6)	87 (38.0)	17 (7.4)	61 (49.6)	55 (44.7)	7 (5.7)	38 (61.3)	22 (35.5)	2 (3.2)	8 (53.3)	3 (20.0)	4 (26.7)	18 (62.1)	7 (24.1)	4 (13.8)	0.01
Broad consent for data-sharing	116 (50.7)	91 (39.7)	22 (9.6)	62 (50.4)	48 (39.0)	13 (10.6)	32 (51.6)	27 (43.6)	3 (4.8)	9 (60.0)	4 (26.7)	2 (13.3)	13 (44.8)	12 (41.4)	4 (13.8)	0.72
Modes of data-sharing	116 (50.7)	101 (44.1)	12 (5.2)	57 (46.3)	59 (48.0)	7 (5.7)	38 (61.3)	22 (35.5)	2 (3.2)	9 (60.0)	5 (33.3)	1 (6.7)	12 (41.4)	15 (51.7)	2 (6.9)	0.47
Data discoverability	102 (44.5)	93 (40.6)	34 (14.9)	51 (41.5)	56 (45.5)	16 (13.0)	28 (45.2)	25 (40.3)	9 (14.5)	7 (46.7)	3 (20.0)	5 (33.3)	16 (55.2)	9 (31.0)	4 (13.8)	0.26
Timeliness of data-sharing	93 (40.6)	109 (47.6)	27 (11.8)	48 (39.0)	56 (45.5)	19 (15.5)	25 (40.3)	33 (53.2)	4 (6.5)	6 (40.0)	9 (60.0)	0 (0.0)	14 (40.3)	11 (37.9)	4 (13.8)	0.34
Cost	66 (28.8)	106 (46.3)	57 (24.9)	31 (25.2)	56 (45.5)	36 (29.3)	19 (30.6)	30 (48.4)	13 (21.0)	7 (46.7)	6 (40.0)	2 (13.3)	9 (31.0)	14 (48.3)	6 (20.7)	0.56

With respect to the field of study, more than 70% of researchers whose primary involvement was clinical research rated as very important only two issues (i.e., ethics and acknowledgment). Those in basic science and laboratory study also rated restriction of use and data access conditions as very important. More than 70% of respondents in social/behavioral study also rated data covered and data documentation as very important. Among those in public health/policy study, more than 70% also rated data documentation as very important.

The only statistically significant difference concerned data availability. For data availability, fewer researchers in clinical and social/behavioral science, compared with the other groups, rated it as very important.

### Perceived challenges in sharing their own research data.

As shown in Table 3, the ratings for perceived challenges tended to be problematic (level 3) or somewhat problematic (level 2). About 40% of respondents rated as highly problematic the top three issues: ethical and legal compliance in sharing individual data, control of the use of “sensitive” or “restricted” data by other researchers, and proprietary data. Only about 20% rated having the necessary resources (time and money) as highly problematic. There were no statistically significant differences when comparing the ratings among researchers in different fields of research work.

**Table 3:** Major concerns about sharing research data (*N* = 229)

Major concerns in data-sharing	Total (*N* = 229)			Clinical (*N* = 123)			Basic science (*N* = 62)			Social/behavioral (*N* = 15)			Public health/policy (*N* = 29)			*P*-value
	Most important, *n* (%)	Important, *n* (%)	Less important, *n* (%)	Most important, *n* (%)	Important, *n* (%)	Less important, *n* (%)	Most important, *n* (%)	Important, *n* (%)	Less important, *n* (%)	Most important, *n* (%)	Important, *n* (%)	Less important, *n* (%)	Most important, *n* (%)	Important, *n* (%)	Less important, *n* (%)	
Issues related to ethical and legal compliance in sharing individual data	106 (46.3)	76 (33.2)	47 (20.5)	57 (46.3)	42 (34.2)	24 (19.5)	30 (48.4)	18 (29.0)	14 (22.6)	7 (46.7)	6 (40.0)	2 (13.3)	12 (41.4)	10 (34.5)	7 (24.1)	0.96
Control of the use of “sensitive” or “restricted” data by other researchers	102 (44.5)	86 (37.6)	41 (17.9)	52 (42.3)	48 (39.0)	23 (18.7)	30 (48.4)	24 (38.7)	8 (12.9)	8 (53.3)	3 (20.0)	4 (26.7)	12 (41.4)	11 (37.9)	6 (20.7)	0.72
Issues related to proprietary data	97 (42.3)	89 (38.9)	43 (18.8)	53 (43.1)	49 (39.8)	21 (17.1)	26 (42.0)	25 (40.3)	11 (17.7)	6 (40.0)	6 (40.0)	3 (20.0)	12 (41.4)	9 (31.0)	8 (27.6)	0.92
Organization/institution policy for data-sharing	70 (30.6)	99 (39.3)	69 (30.1)	39 (31.7)	52 (42.3)	32 (26.0)	20 (32.3)	22 (35.4)	20 (32.3)	5 (33.3)	6 (40.0)	4 (26.7)	6 (20.7)	10 (34.5)	13 (44.8)	0.59
Quality and integrity of sharable data (e.g., complete and homogeneous)	70 (30.6)	82 (35.8)	77 (33.6)	38 (30.9)	48 (39.0)	37 (30.1)	20 (32.3)	22 (35.4)	20 (32.3)	3 (20.0)	4 (26.7)	8 (53.3)	9 (31.0)	8 (27.6)	12 (41.4)	0.59
Organization/institution services or support in performing data-sharing	63 (27.5)	87 (38.0)	79 (34.5)	35 (28.5)	49 (39.8)	39 (31.7)	16 (25.8)	23 (37.1)	23 (37.1)	4 (26.7)	7 (46.6)	4 (26.7)	8 (27.6)	8 (27.6)	13 (44.8)	0.82
Citation of the dataset/original work and acknowledgment of the data repository	59 (25.8)	69 (30.1)	101 (44.1)	34 (27.6)	42 (34.1)	47 (38.2)	13 (21.0)	17 (27.4)	32 (51.6)	4 (26.7)	3 (20.0)	8 (53.3)	8 (27.6)	7 (24.1)	14 (48.2)	0.6
Technical issues (data-sharing platforms, data management, and interoperable systems)	51 (22.3)	106 (46.3)	72 (31.4)	32 (26.0)	62 (50.4)	29 (23.6)	8 (12.9)	30 (48.4)	24 (38.7)	3 (20.0)	4 (26.7)	8 (53.3)	8 (27.6)	10 (34.5)	11 (37.9)	0.054
Necessary resources (time and money)	48 (21.0)	87 (38.0)	94 (41.0)	33 (26.8)	48 (39.0)	42 (34.2)	9 (14.5)	23 (37.1)	30 (48.4)	3 (20.0)	5 (33.3)	7 (46.7)	3 (10.3)	11 (38.0)	15 (51.7)	0.23

## DISCUSSION

We assessed the views of researchers in a middle-income country, Thailand, regarding data-sharing. Previous studies have placed great importance on informed consent and data privacy, identifying specific consent for each new use of data as the major protective mechanism.^6,9,25,27–29^ By contrast, our respondents focused on the importance of safeguards when handling data, including transfer to others, and possible lack of control over how their data are used. Our respondents also rated as very important the need for secondary users to credit the original researchers.

More than 75% of researchers in the fields of clinical and basic science studies and almost 90% of those in social/behavioral and public health/policy studies rated as very important ethical issues in data-sharing. These findings support concerns expressed in the literature. A study in LMICs suggested that an ethical data-sharing practice should be based on four main issues: the value of data-sharing, minimizing harm, promoting fairness and reciprocity, and trust.^30^ A qualitative study by a research group in Thailand on sharing data among stakeholders (research staff, study participants, and community representatives) within its research settings, which may or may not represent Thailand researchers at large, found that the stakeholders generally not only saw benefits in data-sharing but also had reservations about potential harm to research participants, their communities, and the researchers themselves.^29^ Experts in the ethics of human research noted that the issue of data-sharing is often framed as one of individual rights versus societal benefit.^31^ However, some experts postulated that as there are different types of data and different controllers of data; as the technology evolves, the concepts of harm and privacy violations in an era of data-sharing may require rethinking what “public interest” means when data are shared, in contrast with relinquishing traditional rights to privacy.^31^ Further study on this issue is needed as balancing risks and benefits is always challenging not only in research ethics but also in data-sharing ethics.

Despite the concerns about protecting and maintaining the rights, confidentiality, and privacy of the study participants, data-sharing should be developed and work within and around any legal requirements.^32^ Almost 50% of our respondents raised concerns about legal compliance in sharing personal data. Regulations in Thailand and other countries clearly define data containing personal identifiers and anonymous data, and usually dictate restrictive policies on the use of the data because of privacy concerns.^14,25^ A study that reviewed data access from the national databases in Thailand and other countries in the Asia–Pacific region also noted that the issues of data-sharing in Thailand and other Asia–Pacific countries have been compounded by the issue of privacy protection such that researchers and academics can access the data files only through certain application processes, which are sometimes unclear and complicated.^25^ Recently in Thailand, the Personal Data Protection Act, which is quite similar to the European GDPR, has made an impact on the review of research proposals; many RECs at the institutional level now stress the importance of clear data-sharing processes in submitted research proposals, covering what/when/where/how the data would be shared, either as primary data producer or secondary data user.

In sharing data, even when the data are de-identified and shared, some may view it as an invasion of privacy and a source of potential risk.^4,9,33,34^ The provision of informed consent by study participants for the future research use of data or bio-specimens is thus required. The informed consent process can use different approaches, such as blanket, broad, checklist, and study specific; each format will impact on how the data or specimens will be shared in the future.^5,35,36^ Council for International Organizations of Medical Sciences and other guidelines usually suggest the use of broad consent for data-sharing purposes; this is less specific than consent for each use, but narrower than blanket consent.^5,37–39^ However, an empirical study showed that providing information on data-sharing and obtaining broad consent for data-sharing, in addition to consent for the primary study, made the consent process more complex and difficult to comprehend by the study participants, particularly when the study was conducted in rural areas of LMICs.^40^ A qualitative study among stakeholders of a research unit based in Thailand demonstrated that clinical-trial participants mainly focused on information about the potential benefits and harms of data-sharing and how much information should be provided about data-sharing.^28^ It is important to have effective, valid, consent process. Broad consent could be valid if there was some clarity at the time of consent, the kinds of people, or institutions to be shared with, and how, in broad terms, the data would likely be used.^28,29^ In this study, only about half of the researchers rated the use of broad consent in acquiring data for sharing as very important. It is postulated that many researchers might have usually applied broad consent in their studies. This issue needs further investigation.

Another issue related to control was ranked more important than informed consent. Our respondents indicated that researchers should decide what types of project data are shareable and which ones are likely to be useful to the scientific community. They also thought that it is important for researchers to have plans that outline the conditions under which other researchers can access and reuse data. One example of data-sharing condition was seen in the findings of a qualitative study among stakeholders in a research unit in Thailand such that very few researchers were in favor of having the entire data set, including unpublished data, publicly available without any controls, whereas almost all were in favor of making data on which publications were based publicly accessible.^29^ Another study of an ongoing trial under NIH (United States-NIH) support reported that the decision to release data over the years was determined and performed by the primary researchers based on periodical trend analysis.^27^

Another example of control over data-sharing, as noted in the literature, was the restriction of use where it may be related to sociocultural context and trust. Trust between a data producer/provider and data user greatly enables data-sharing, whereas the absence of trust would make providers cautious about potential data misinterpretation, misuse, or intentional abuse.^9,33,41^ Although not statistically significant, more than 70% of researchers working in basic science and laboratory, more than 60% working in other clinical and social science studies, and more than 50% in public health studies rated the restriction of use and data access as very important. These results correspond with the second top rating for researchers’ concerns about controlling the use of “restricted” or “sensitive” data by other researchers. The proportion of researchers in basic science rating restriction of data access and use highly may be due to the perception that data from basic science studies are more likely to be used as the basis for further studies than data from other types of studies. Although research funders, regulators, and journals request researchers share individual-level health data, most researchers, particularly in LMICs, have no guidelines or policies to guide them about data restriction and sensitivity.^10^ Clear policies covering the control of data access are needed. The terms of access that should be considered include the following: which institutions and researchers are allowed access; which research projects should be supported, is the benefit shared with communities; where should the repository be located, who operates the facility; who uses the data, who gets what benefits; and what regulations apply.^2,4,42^ However, as discussed in the literature of evolving information technologies, in the future, data-sharing may eventually become quite common and considered a low-risk activity deserving only a limited amount of procedural scrutiny.^34,43^ A study on the challenge of equitable data-sharing in multi-countries, including Thailand, suggested that international guidelines should be revised such that researchers or data producers should obtain consent for sharing their data with secondary users. However, there should be clear definitions of sensitive data to mitigate any potential harm to data subjects and their communities.^27^ This article also recommended the promotion of data-sharing and that research groups and institutions should establish their own data-sharing policies tailored to their context, data, and community, while remaining harmonized with existing policies as far as possible.

Even though our respondents wanted to place conditions on data access, they believed that data-sharing is important. They felt it was important that researchers provide data from their study sufficient to reproduce analysis of the primary outcomes. However, this remained a challenging issue because there was no common data-sharing platform or framework in place in the country. A research group based in Thailand examined the establishment of a data-sharing policy and DAC; the team noted that the existence of a data management and data-sharing policy is the first and vital step in encouraging researchers and other data producers to share their data.^10^ A qualitative study by this group also reported that many stakeholders preferred a governance committee or trusted gatekeeper to oversee requests for appropriate data access and use.^29^ They proposed that DACs should not be modeled on RECs because of their different functions and goals of review; DACs would conduct reviews based on the principles of public health ethics, whereas RECs focused on research ethics.^44^ Although many RECs in Thailand request that researchers include a data-sharing section in their proposals, according to the recommendations of funding agencies and CIOMS,^5,42^ the authors of this study support the implementation of DACs, at least at the institutional level, to review and assist in effectively and efficiently accessing and using secondary data. As stated in the literature that there is no widely accepted framework and functions,^44^ thus, DACs should be established according to the institutional and legal frameworks of the country, while taking into account the requirements and common practices in data-sharing proposed and enforced by several international ethical guidelines, funding agencies, and journal editors.^5,42,45^ As part of data-sharing frameworks, the DACs’ operating procedures may be adapted from existing procedures for accessing national databases, including, for example, data access agreements, data transfer process, and data security and protection.

Specifically, respondents endorsed the importance of reciprocity and indicated that data-sharing practices have not always been fair. They also expressed concern that data producers tended to receive little credit or benefit from their work, whereas data users benefit, academically and/or commercially, from it.^1,9^ In sharing the data of publication-related data/materials, many journal editors have also raised concerns that data are frequently not made available for sharing by the primary researchers, and many secondary users did not cite the original data sources they used.^46^ Regarding sharing their own research data, about 25% of our respondents noted concern about the citation of the dataset and original work, and acknowledgment of the data repository. Others have argued that protection of subjects requires that the researchers control the specific studies for which their data are used. By contrast, our respondents felt that it is important to place conditions on the transfer of data. These conditions, rather than the control over what happens to the samples by the data subjects, should protect the interests of both the data subjects, the original researchers, and the usefulness of the data for new research.

Related to acknowledgment in using data, about 40% of researchers rated concerns about proprietary data issues highly. As suggested in the literature, a lack of clarity about ownership rights and intellectual property issues may make it difficult to determine who has the authority to decide how data should be shared.^1^ As part of the data-sharing agreement, intellectual property rights should be defined describing the entities or persons who will hold the intellectual property rights to the data and how intellectual property will be protected, if necessary.^41^ It is thus important that mechanisms exist to recognize the intellectual property of the primary researchers for producing and curating data sets for sharing. Ownership and property right can be used to restrict rather than extend data access.^9^ This issue might be clarified by having a widely accepted data-sharing policy under the management and control of DACs established at institutional or national levels.

Interestingly, only about 20% of researchers rated concerns for dealing with technical issues and having the necessary resources (time and money) highly. This corresponds with fewer researchers’ rating the cost of data-sharing as very important. In fact, in sharing data, the researchers are required to prepare and submit not only data but also metadata (document that describes data content, origin, methods, etc.) and potentially other documentation related to the data (e.g., protocol, case record forms, data edit specification, and others). In the stated data-sharing requirements of journal editors, it is suggested that the following should be described: whether individual de-identified participant data will be shared; what data in particular will be shared; whether additional, related documents will be available; when the data will become available and for how long; and by what access criteria.^6^ Fulfilling these obligations requires effort, resources, and an efficient data management team. Without a data management team, the process might be quite cumbersome, as shown in a study on data collected in healthcare databases in Thailand and other Asia–Pacific countries, where researchers confronted issues of data quality and standardization when they sought to extract and merge data from fragmented databases and systems.^25^ Data use and sharing depend on the existence of a functioning technological infrastructure and interoperability of health IT systems; however, solutions are available.^47^ The process of data-sharing requires human and technical resources for data preparation, annotation, communication with recipients, computer equipment, and internet connectivity; researchers must therefore invest time and effort in data collection and sharing.^9,38^ As reported in one study, investment in sharing data has economic implications and the motivation to share and the investment made to do so do not yield an immediate return; this may be one reason for the reluctance of many researchers to share their data.^7^ Other studies in LMICs have stressed the importance of capacity building and investment in data management and data science skills, as well as in data-sharing platforms.^27,29,40^ In the present study, the researchers may not have been widely aware of the requirement for data-sharing or prepared the resources necessary to do so, or did not fully comprehend what data-sharing entails. This could be one barrier inhibiting data-sharing in Thailand and possibly other LMICs. To promote data-sharing, investment in data management and platforms is required, together with the establishment of DACs, at least at the institutional level, to assist researchers manage the required data-sharing activities.

### Limitations of the study.

The overall response rate was very low. As mentioned in the Data Collection section, both the paper-based and online versions of the survey were distributed to 218 workshop participants from 38 academic/health-science institutes and the online version to 124 university hospitals/research institutes and alumni of FTM. It should be noted that, among those 218 workshop participants, most answered online, whereas only a few answered the hard copy questionnaire. A total of 2,656 e-mails with links to the questionnaire were sent out, and the wait time for returned responses was set at 4 months. One limitation of this online survey was that there were no reminder emails. Another limitation of this online survey was that the database collecting the returned online questionnaires could not identify whether the responses emanated from workshop participants or from researchers who received the links at different institutions. This may have biased the results. In addition, most of the surveys were completed online and the views of individuals who lacked access to the internet may have differed. This concern may be minimal for the present survey, given that the target respondents were researchers who worked in academic institutes and who generally had access to the internet as an integral part of their work. Numerous other reasons besides internet access may have prevented a higher response rate. Because the low response rate could increase the uncertainty of the results, although the questionnaires were distributed to almost all academic/research institutes in Thailand, the interpretation of the study results could be biased as the respondents may or may not be representative of researchers in Thailand. In addition, the respondents might not be representative of all research fields; most of the respondents worked in clinical and basic science studies and only a few in social/behavioral and public health/policy studies. Readers should thus exercise caution in generalizing the study results.

## CONCLUSION

Data-sharing is an effective way to advance scientific research and to assure that the benefits derived from research data are realized as widely as possible. Previous work has pointed to the ethical challenges faced by this effort and raised concerns that these challenges may be especially difficult in LMICs. Our respondents, researchers in Thailand, expressed lower levels of concern regarding informed consent and the feasibility of conducting research and sharing data. They were more concerned with the importance of appropriate acknowledgment and protecting the legal rights of the primary data collectors and providers. The implications of these results are important for future efforts to include LMICs in data-sharing frameworks. They highlight the importance for the sustainability and fairness of these efforts to ensure that parties in LMICs receive sufficient credit and are involved in determining the studies in which their collected data are used. To promote data-sharing, investment is required in the development of data-sharing platforms and data management skills, together with the establishment of DACs, at least at the institutional level.

## Supplemental questionnaire file

Supplemental materials
