# Supplementary material for: Issues and Challenges Associated with Data-Sharing in LMICs: Perspectives of Researchers in Thailand
Source: Am J Trop Med Hyg. 2020 May 11;103(1):528–36. doi: 10.4269/ajtmh.19-0651 (PMC7356467; doi:10.4269/ajtmh.19-0651)
Supplement: Supplementary file 1 [file tpmd190651.SD1.pdf]

<u>แบบสอบถามความเห็นเกี่ยวกับประเด็นเชิงวิพากษ์ด้านการวิจัยทางสุขภาพ</u>

Survey on Critical and Controversial Issues in Health Research

แบบสอบถามนี้ มีวัตถุประสงค์เพื่อสำรวจความคิดเห็นของนักวิจัยด้านชีวการแพทย์ สาธารณสุข สังคมศาสตร์ พฤติกรรมศาสตร์ ในประเด็นที่ยังคงเป็นข้อวิพากษ์กันอยู่ในปัจจุบัน คือ **การแบ่งปันข้อมูล (data sharing)** โปรดตอบแบบสอบถามนี้ เพื่อคณะผู้จัดทำจะสามารถวิเคราะห์ความคิดเห็นของท่านต่อประเด็นเหล่านี้ เป็นการแลกเปลี่ยนเรียนรู้ และเป็นประโยชน์ต่อวงการวิชาการต่อไป อย่างไรก็ดี ท่านสามารถข้ามข้อที่ไม่ต้องการตอบโดยที่ไม่มีข้อผูกมัดใดๆ และขอรับรองว่าข้อมูลจากแบบสอบถามของท่านจะเป็นความลับและไม่สามารถระบุได้ว่าใครเป็นผู้ตอบ
ขอขอบพระคุณท่านเป็นอย่างสูงสำหรับความร่วมมือ

This survey aims to examine the opinions of researchers in the biomedical, public health and social science/behavioral sciences about current critical but controversial issues. This questionnaire is focused on **data sharing** topic. Please complete the questionnaire to help us understand researchers' opinions on these matters. Please skip any item you do not wish to answer. This survey is guaranteed anonymous.

Thank you very much in advance for your kind responses.

<u>ข้อมูลพื้นฐาน (</u>Demographic data)

เพศ: ____ ชาย ____ หญิง

Sex: ___ Male ___ Female

ลักษณะงานวิจัยหลักของท่าน:

Main research field:

___ งานวิจัยทางคลินิก (Clinical study)

___ งานวิจัยทางชีวการแพทย์ / ห้องปฏิบัติการวิจัย (Biomedical/Laboratory Study)

___ งานวิจัยทางสาธารณสุข / งานวิจัยเชิงนโยบาย (Public Health/Policy Research)

___ งานวิจัยทางสังคมศาสตร์ / งานวิจัยทางพฤติกรรมศาสตร์ (Social Science/Behavioral Research)

___ อื่นๆ โปรดระบุ (Other please specify) ___________________

จำนวนปีที่ทำงาน: _____1-3 ปี ___ 4-6 ปี ___ 7-10 ปี ___ 11-15 ปี ___ มากกว่า 15 ปี

Years working in research field:

____ 1-3 years ____ 4-6 years ____ 7-10 years ____ 11-15 years ____ more than 15 years

ประเด็นเรื่อง การแบ่งปันข้อมูล (Data Sharing)

คำจำกัดความ:

Definitions:

"ข้อมูลจากการวิจัย (Research Data)" ที่จะถูกนำมาแบ่งปันกันนั้น มักจะแตกต่างกันไปแล้วแต่สาขาของการวิจัย แต่ส่วนมากจะหมายถึง ผลลัพธ์จากการศึกษา หรือจากการทดลอง ที่ทำให้สามารถพิสูจน์ผลการวิจัยนั้นๆ ได้หรือเป็นข้อมูลที่เกี่ยวข้องกับโครงการวิจัย เช่น ข้อมูลดิบ หรือข้อมูลที่ประมวลผลแล้ว ซอฟต์แวร์ (software) รหัสโปรแกรม (code) โมเดล (models) อัลกอริธึม (algorithms) โครงร่างวิจัย (protocols) หรือระเบียบวิธีวิจัย ตัวอย่างของข้อมูลจากการวิจัยที่แตกต่างกันไป เช่น ข้อมูลที่เป็นภาพ (imaging data) ข้อมูลทางพันธุกรรม (genotypic data) ข้อมูลทางคลินิก (clinical data) และข้อมูลจากแบบสอบถาม เป็นต้น

“Research Data” sharing often differs per field, but generally refers to the results of observations or experiments that validate your research findings and materials associated with your research project, including: raw or processed data files, software, code, models, algorithms, protocols, and methods of your study. Examples of different data types may include imaging data; genotypic data; clinical measurements; and data generated from surveys.

วัตถุประสงค์หลักของการแบ่งปันข้อมูล เป็นไปเพื่อสนับสนุนให้เกิดการพัฒนาทางวิชาการอย่างต่อเนื่อง และเป็นการใช้ผลการศึกษาให้ได้ประโยชน์สูงสุด

The main purpose of “**Data-Sharing**” is to support good science and to get the most of our research investments.

วิธีตอบคำถาม:

Instructions:

โปรดให้คะแนนความสำคัญของหัวข้อต่างๆ ต่อไปนี้ (คะแนนความสำคัญเรียงลำดับจากน้อยไปมาก คือ 1 = สำคัญน้อยที่สุด / 2 = สำคัญน้อย / 3 = สำคัญ / 4 = สำคัญมากที่สุด)

Please rate your own importance levels for the following items. [1=less important, 2=somewhat important 3=important, 4=very important]

หัวข้อ	ความหมาย	1	2	3	4
**การปกปิดข้อมูล**<br>(Data covered)	นักวิจัยสามารถตัดสินใจได้ว่า ข้อมูลแบบไหนทีควรถูกนำมาแบ่งปันและน่าจะเป็นประโยชน์ต่อวงการวิทยาศาสตร์<br>Researchers can decide what types of project data are shareable and which ones are likely to be useful to the scientific community.				
**ข้อจำกัดในการใช้ข้อมูล**<br>(Restriction of use)	นักวิจัยควรควบคุมข้อจำกัดในการเข้าถึงข้อมูลได้ด้วยเงือนไขทีชัดเจน และนักวิจัยสามารถจะควบคุมการเปิดเผยข้อมูล หากมีประเด็นเรื่องทรัพย์สินทางปัญญา<br>Researchers may be able to regulate access to their data, and/or control access to data in order to protect intellectual property (if any).				
**ความยินยอมแบบเปิดกว้างสำหรับการแบ่งปันข้อมูล** (Broad consent for data sharing)	ในกระบวนการขอความยินยอมของการวิจัย นักวิจัยควรจะขอความยินยอมเกี่ยวกับการนำข้อมูลไปแบ่งปันใช้กับการวิจัยอื่นในอนาคต (Broad consent)<br>Regarding consent procedures, researchers should inform their				

หัวข้อ	ความหมาย	1	2	3	4
	study participants about future use of data.				
**รูปแบบของการแบ่งปันข้อมูล** (Modes of data sharing)	นักวิจัยสามารถเลือกรูปแบบของการจัดเก็บ และการเข้าถึงข้อมูล เช่น การจัดเก็บในคลังข้อมูลกลาง (archived database) หรือคลังข้อมูลที่มีการควบคุมและจำกัดการใช้งาน (enclave database) Researchers can select various secure methods for data storage and access such as archived, enclave database.				
**ข้อมูลอ้างอิง** (Data documentation)	นักวิจัยควรจะให้คำอธิบายทีชัดเจนเกียวกับข้อมูลทีจัดเก็บในงานวิจัย (metadata) ควบคู่กับข้อมูลจากงานวิจัยที่จะนำมาเปิดเผย Researchers should share metadata (information about the study data) together with the data to be shared.				
**ความสามารถในการประยุกต์ใช้ข้อมูล** (Data discoverability)	นักวิจัยควรจะเตรียมข้อมูลให้ครบถ้วนพร้อมใช้งาน เพื่อให้นักวิจัยอื่นนำไปประยุกต์ใช้และพัฒนาองค์ความรู้ใหม่ Researchers should make data available for further discoveries by other researchers.				
**เงื่อนไขในการเข้าถึงข้อมูล** (Data access conditions)	นักวิจัยควรจะกำหนดกฎเกณฑ์การเข้าถึงข้อมูล และการนำข้อมูลกลับมาใช้ใหม่ Researchers should have plans that outline the conditions under which other researchers can access and re-use data.				

หัวข้อ	ความหมาย	1	2	3	4
**การแบ่งปันข้อมูลที่พร้อมให้พิสูจน์** (Data availability)	นักวิจัยควรจะให้ข้อมูลการวิจัยของตนให้พอเพียง เพื่อให้ผู้อื่นสามารถพิสูจน์ผลการศึกษาได้<br>Researchers should provide data from their study sufficient to reproduce the analysis of the primary outcomes.				
**แบ่งปันข้อมูลได้ในเวลาที่เหมาะสม** (Timeliness of data sharing)	นักวิจัยควรแบ่งปันข้อมูลในเวลาที่เหมาะสมเพื่อให้ได้ประโยชน์สูงสุด<br>With respect to the value of data, researchers should release and share data on a well-timed basis.				
**ประเด็นเรื่องจริยธรรม** (Ethical issues)	นักวิจัยต้องมีมาตรการควบคุมระหว่างความเป็นส่วนตัว ความปลอดภัย และความมั่นคงของข้อมูลของผู้ร่วมวิจัย กับผลประโยชน์เชิงพาณิชย์จากการใช้ข้อมูลของผู้ร่วมวิจัยนั้น<br>Researchers must have protective procedures balancing between privacy / safety of studied participants and potential commercial interests.				
**ค่าใช้จ่าย** (Cost)	ข้อมูลทั้งหมดที่ถูกแบ่งปัน ควรเป็นการให้ฟรี หรือมีค่าใช้จ่ายที่ไม่มากเกินกว่าค่าใช้จ่ายในการบริหารจัดการในการทำสำเนาข้อมูล<br>All shared data should be free of charge or at no greater cost than the costs of reproduction/ dissemination.				
**การแสดงความขอบคุณ**	ผู้ที่นำข้อมูลไปใช้ ควรอ้างอิงและให้เครดิตแก่เจ้าของข้อมูล				

หัวข้อ	ความหมาย	1	2	3	4
(Acknowledgement)	Secondary users of data should credit the original researcher(s).				

หากท่านต้องแบ่งปันข้อมูล ท่านจะรู้สึกเป็นปัญหามากน้อยเพียงใด ในเรื่องต่อไปนี้ (คะแนนระดับความยาก ง่าย เรียงจากน้อยไปหามาก คือ 1 = ง่าย / 2 = ค่อนข้างยาก / 3 = ยาก/ 4 = ยากมาก)

If you have to share your research data, what would be the levels of difficulty related to the following issues? [1=none, 2=somewhat problematic, 3=problematic, 4=highly problematic]

**เรื่องทีทำให้นักวิจัยรู้สึกยาก และรู้สึกเป็นภาระ หากต้องมีการแบ่งปันข้อมูล**<br>**Difficulties and burdens faced by researchers**	1	2	3	4
ทรัพยากรที่จำเป็น เช่น เวลา/เงิน<br>Necessary resources (time, money)				
ประเด็นทางเทคนิค เช่นรูปแบบของการแบ่งปันข้อมูล / การจัดการข้อมูล / ระบบปฏิบัติการ (interoperable systems)<br>Technical issues (data sharing platforms, data management, interoperable systems)				
ประเด็นเรืองเกียวกับกรรมสิทธิของข้อมูล<br>Issues related to proprietary data				
ประเด็นเชิงจริยธรรมและข้อกฎหมายในการแบ่งปันข้อมูลส่วนบุคคล				

เรื่องที่ทำให้นักวิจัยรู้สึกยาก และรู้สึกเป็นภาระ หากต้องมีการแบ่งปันข้อมูล<br>Difficulties and burdens faced by researchers	1	2	3	4
Issues related to ethical and legal compliance in sharing individual data				
นโยบายขององค์กร / สถาบันของท่าน ต่อการแบ่งปันข้อมูล<br>Organizational/institutional policies for data sharing				
ความช่วยเหลือ หรือการสนับสนุน จากองค์กร / สถาบันของท่าน ต่อการจัดให้มีการแบ่งปันข้อมูล<br>Organizational/institutional services or supports to perform data sharing				
คุณภาพและจรรยาบรรณของข้อมูลที่แบ่งปัน (ความสมบูรณ์ของข้อมูล / แบบแผนเดียวกัน)<br>Quality and integrity of shareable data (e.g. complete, homogeneous)				
การควบคุมนักวิจัยผู้อื่นในการใช้ข้อมูลที่ "อ่อนไหว" หรือ "มีข้อจำกัดในการใช้งาน"<br>Control of the use of 'sensitive' or 'restricted' data by other researchers				

**เรื่องทีทำให้นักวิจัยรู้สึกยาก และรู้สึกเป็นภาระหากต้องมีการแบ่งปันข้อมูล**<br>**Difficulties and burdens faced by researchers**	1	2	3	4
การอ้างอิงถึงข้อมูลต้นฉบับ และกิตติกรรมประกาศของฐานข้อมูล<br>Citation of the dataset (original work) and acknowledgement of the data repository				

ความคิดเห็นเพิ่มเติมในเรื่องการแบ่งปันข้อมูล (Other thoughts/comments about data sharing:)

____

“ขอขอบพระคุณที่ท่านได้สละเวลาตอบแบบสอบถามชุดนี้”

Thank you very much for your time and attention.
